# From Deep-Level Similarity to Subordinate *Moqi*: The Mediating Role of Leader-Member Exchange

**DOI:** 10.3389/fpsyg.2022.879284

**Published:** 2022-05-11

**Authors:** Lan Li, Xingshan Zheng, Siwei Sun

**Affiliations:** ^1^Shanghai Business School, Shanghai, China; ^2^Organization Management Department, Antai College of Economics & Management, Shanghai Jiao Tong University, Shanghai, China; ^3^Department of Management and International Business, Business School, The University of Auckland, Auckland, New Zealand

**Keywords:** deep-level similarity, leader-member exchange, subordinate *moqi*, self-categorization theory, similarity

## Abstract

Fostering subordinate *moqi* is a significant method to improve the cooperating quality and promote positive outcomes. However, little is known about the influencing factors and mechanisms of subordinate *moqi*. To address this issue and explore the influencing factors of subordinate *moqi*, we draw on self-categorization theory to develop a mediation model to examine whether and how deep-level similarity affects subordinate *moqi*, casting the leader-member exchange (LMX) as a mediator. A two-wave online survey was conducted and 316 data was collected. A Structure Equation Modeling analysis was used to test all hypotheses with Mplus 7. Results showed that the deep-level similarity could positively predict subordinate *moqi* and LMX, respectively. Additionally, LMX was a significant predictor of subordinate *moqi* as well as it mediated the positive relationships between deep-level similarity and subordinate *moqi*. These findings expand our understanding of the antecedents of subordinate *moqi*. It is suggested that developing subordinates’ deep-level similarity with supervisors and LMX relationships are instrumental in cultivating subordinate *moqi* which promote positive outcomes. Organizations should integrate effective management programs into managerial strategies to enhance deep-level similarity and LMX, in turn, fosters subordinate *moqi*.

## Introduction

*Moqi*, which comes from a Chinese poem of the Song Dynasty, refers to a state of unspoken but tacit understanding between two parties (Zheng et al., 2019a). It is a construct cultivated by Eastern culture (i.e., high context, face, and power distance) (Zheng et al., 2019a). The importance of *moqi* is argued by Zhang and Zhang (2011) as being based on the need to understand consistently the implicit information of the interactive process. To keep consistency in the understanding of implicit information between implicit information conveyers and receivers, people need to build *moqi*. Especially in high context, face culture, and high power distance societies (e.g., China), much implicit information is involved (Zheng et al., 2019a).

In high context, people generally involve indirect expression (i.e., non-verbal messaging) to convey information to others (Richardson and Smith, 2007). Namely, they convey clues through their eyes, gestures, tone, expressions, and so on. Only if people, who are information receivers, understand exactly the means of this non-verbal information they can be regarded as getting complete information and reduce mistakes. Secondly, face reflects individuals’ self-image from social recognition and reputation obtained in their life (Ho, 1976). In the face culture context, individuals are more eager to get face and do not lose face (Zhang et al., 2011). Thus, individuals desiring for face may adjust their behavior when they encounter face risk (Kim and Sang, 1998). For example, when individuals infer that their thoughts may make themselves or others very embarrassing and awkward; individuals are reluctant to express their real thoughts directly to save face (Bao et al., 2003). In this context, they may convey their real thoughts through their expression, eyes, gestures, tones, speed of delivery, and so on and expect others to understand their thoughts unspoken as well. Additionally, power distance is the third prominent culture related to *moqi* and refers to “the extent to which a society accepts the fact that power in institutions and organizations is distributed unequally” (Hofstede and Bond, 1984, p. 419). High power distance culture is salient in China (Hofstede and Bond, 1984). Influenced by the culture of high power distance, leaders generally have strong authority and do not say everything thoroughly and clearly when arranging tasks for their subordinates, meanwhile subordinates are also afraid of authority or do not want to encounter embarrassment, and so dare not or do not want to ask their leaders more (Liao H. et al., 2010; Zhang et al., 2011). In these contexts, mentioned above, *moqi* is an effective solution for helping people to understand messages unspoken and likewise protect people’s authority and face (Zheng et al., 2019a).

Although *moqi* is important in the Chinese cultural context, the term *moqi* is only widely used in people’s daily life (Zhang and Zhang, 2011). It was not until Zheng et al. (2019a) introduced *moqi* into the field of organizational behavior research that it was developed as an academic concept. They focused on *moqi* between subordinates and supervisors and proposed the construct of subordinate *moqi*, which reflects a state that subordinates can fully and correctly know their supervisors’ desires, intentions, and expectations, even if supervisors do not talk with them directly (Zheng et al., 2019a). They developed the scale of subordinate *moqi* and conducted empirical research to investigate the effects of *moqi*. Their findings show that subordinate *moqi* significantly is associated with employee task performance (Zheng et al., 2019a). Additionally, more and more researchers have focused on the research of *moqi* in recent years and they found subordinate *moqi* is related to positive outcomes. For example, previous studies show that subordinate *moqi* can decrease knowledge hiding (Zhong et al., 2021), and is a significant predictor of knowledge sharing behavior (Zheng et al., 2019b), employee voice (Zhou and Zheng, 2019), empowerment (Li et al., 2020), and work engagement (Li and Zheng, 2020). The positive effects of subordinate *moqi* make us believe that it is an important concept to know. More researches should be called for exploring subordinate *moqi*.

Today, facing the heavy work brought by the increasingly competitive market, managers always cooperate with their subordinates to handle the tasks (Fisher et al., 2012). To improve the quality of cooperation and achieve positive outcomes, organizations encourage employees to build subordinate *moqi* (Zhang and Zhang, 2011; Wang et al., 2018; Zheng et al., 2019a). However, it is not easy to establish this state (i.e., subordinate *moqi)* since little is known about the antecedents of subordinate *moqi.* Till now, only several studies explored the influencing factors of subordinate *moqi.* One of the works took an information-seeking approach (Zheng et al., 2019a), and the other adopted a proactive motivation model (Wang et al., 2018) to explore antecedents of subordinate *moqi.* They found that implicit/explicit feedback-seeking and perceived supervisory status were two essential predictors of subordinate *moqi*. Additionally, Zhong et al. (2021) found that leader humility is positively related to subordinate *moqi*. These studies from a “subordinate-centric” and “leader-centric” perspective have provided valuable insights into *what* influences subordinate *moq*i; however, little is known about what may influence subordinate *moqi* from the perspective of “subordinate-supervisor.” Since that subordinate *moqi* is imperative in the workplace, it is critical to explore *how* to cultivate subordinate *moqi*.

Thus, we aim to explore the influencing factors of subordinate *moqi* from the perspective of “subordinate-supervisor” to fill this gap and enable managers to understand how to foster subordinate *moqi*, which is conducive for task performance (Zheng et al., 2019a) and positive employee behaviors (Zhou and Zheng, 2019). Drawing on self-categorization theory (Haslam and Reicher, 2015), this study develops a mediation model to explain *whether* and *how* deep-level similarity influences subordinate *moqi*.

Subordinate *moqi* links subordinates and supervisors (Wang et al., 2018). If those factors from the “subordinate-centric” perspective will affect subordinate *moqi*, we infer that the common characteristics between subordinates and supervisors, from the perspective of “subordinate-supervisor,” may also affect subordinate *moqi*. Deep-level similarity between subordinates and supervisors is a salient concept that indicates subordinates and supervisors’ consistency in belief, value, and attitude (Ndiaye, 2011), outlook, perspective, and problem-solving (Abu Bakar and McCann, 2018). These characteristics significantly affect individuals’ consistency in judgments, decisions, and actions on issues or goals (Ndiaye, 2011). In other words, deep-level similarity between subordinates and supervisors may enhance cognitive and action consistency (Deng et al., 2014) which is related to subordinate *moqi* (Zhang and Zhang, 2011). This clue suggests that deep-level similarity between subordinates and supervisors may be associated with subordinate *moqi*. Drawing on the self-categorization theory (Haslam and Reicher, 2015), subordinates may be stimulated to conduct self-categorization behavior by their deep-level similarity with their supervisors. The categorization results will help employees distinguish whether they belong to the same category as their supervisors or not. If subordinates and supervisors perceive that they belong to the same category because of deep-level similarity, they are more likely to attain the same recognition, and then reach consensus on the goals and behaviors (Graziano et al., 2007). Since subordinate *moqi* embodies “consensus intention,” which reflects that subordinates and their supervisors have consistent understandings of goals, it may inspire employees to spontaneously choose consistent behavior (Zhang and Zhang, 2011). Therefore, we argue that employees’ deep-level similarity with supervisors may play a vital role in promoting the achievement of subordinate *moqi*.

Additionally, we put forward one possible mediating mechanism between deep-level similarities and subordinate *moqi* according to the self-categorization theory. Based on the self-categorization theory, deep-level similarity will induce a categorizing process that initiates in-groups and out-groups (Oldmeadow et al., 2003). And then individuals with a high level of similarity are more likely to be in-groups and develop high-quality relationships (Chen et al., 2008). When employees and their supervisors develop this relationship, they may increase the frequency and quality of interactions (Graen and Uhl-Bien, 1995). The quality of leader-member exchange (LMX) will be improved. Therefore, subordinates may become more familiar with their supervisors and know supervisors’ unspoken desires and ideas (i.e., subordinate *moqi*). Meanwhile, subordinates who are out-group members will try to get closer to the supervisors to get positive feedbacks from the high ranking. Thus, they will increase the frequency of communication with supervisors to achieve further familiarity between them and supervisors. Thus, we propose that the LMX relationship is one possible underlying mechanism that explains the relationship between deep-level similarities and subordinate *moqi*.

Our research can make three theoretical contributions. First, we contribute to the scholarly conversations around antecedents of subordinate *moqi* from a new viewpoint. To better comprehend the influencing factors of subordinate *moqi*, we posit that deep-level similarity between subordinates and supervisors can promote subordinate *moqi* from a dyadic perspective. Namely, we explore the antecedents of subordinate *moqi* from the subordinate-supervisor dyadic perspective rather than viewing subordinate *moqi* as a result of subordinates themselves from the subordinate-centric perspective. This change extends the scope of theorizing antecedents of subordinate *moqi* and provides a new perspective of exploring mechanisms of initiation of subordinate *moqi.*

Additionally, we regard the LMX relationship as a possible underlying mechanism through which deep-level similarity can influence subordinate *moqi*. Research suggests that when employees have deep-level similarities with their supervisors, they will develop high-quality leader-member exchange relationships (Sherony and Green, 2002; Valenzuela et al., 2020) and indirectly generate subordinate *moqi* (Casimir et al., 2014; Srivastava and Dhar, 2016). Our study provides a possible path to explain how dyadic traits between subordinates and supervisors lead to subordinate *moqi*.

Finally, this study extends the perspective of exploring followership. Compared with previous studies which focus on the angel of leaders or leadership to discuss the influencing factors of followers’ behaviors, this study, from a subordinate-supervisor dyadic perspective, explores the influence of the traits linking subordinates and supervisors on followers’ behavior.

## Theoretical Framework

### Subordinate *Moqi*

Subordinate *moqi* reflects a state in which subordinates understand supervisors or have silent consensuses with supervisors without verbal communication (Zheng et al., 2019a). The stronger subordinate *moqi* means the better subordinates understand their supervisors. When supervisors imply something in a non-verbal way, subordinates can tacitly perceive and understand it and then conduct consistent actions in their supervisors’ expectations (Zheng et al., 2019a). During the *moqi* generating process, supervisors are information transmitters, while subordinates are information receivers and interpreters. Why can one party tacitly comprehend the other party without speaking? Some scholars believe that traditions, norms, and assumptions of socio-cultural language are the sources of the consistent actions between two parties (Yao and Chen, 2017).

Zhang and Zhang (2011) believe that keeping consistent with supervisors in intrinsic ideology is good for subordinates to achieve consistent understanding and actions toward the goals. Some studies explore the antecedents of subordinate *moqi* from the perspective of subordinate-center. Recent empirical research found that subordinates’ explicit and implicit feedback-seeking were two significant predictors of subordinate *moqi* (Zheng et al., 2019a). Besides, Wang et al. (2018) found that employees’ perceived supervisory status, as one antecedent of subordinate *moqi*, positively influenced subordinate *moqi* via feedback-seeking. Additionally, from the perspective of “leader-center,” Zhong et al. (2021) provided evidence that leader humility can significantly predict subordinate *moqi* by an empirical research. Subordinate *moqi* links employees and their supervisors. Accordingly, we infer that from the perspective of the link between subordinate and supervisor, factors related to subordinate-supervisor traits may affect subordinate *moqi*, such as employees’ deep-level similarity with supervisors. The similarity is one important term when we discuss the interaction between subordinate and supervisor, especially in discussing self-categorization (Hogg and Terry, 2000; Zhao et al., 2014). However, it is not clear whether or how employees’ deep-level similarity with supervisors triggers subordinate *moqi.* Thus, this study aims to discuss the relationships between deep-level similarity and subordinate *moqi.*

### Self-Categorization Theory

The self-categorization theory developed from the social identity theory assumes that the comparison between one party and other parties in related social activities triggers the functioning of the social self-concept (Oldmeadow et al., 2003). Generally, there are three comparison levels of self-categorization. The theory believes that individuals are human beings that differ from other creatures. Subsequently, individuals can be insiders or outsiders of groups according to their race, nation, or others. Finally, an individual is a unique unit that differs from others in groups because of the differences in personality or other forms (Haslam and Reicher, 2015). The comparison makes individuals form into different clusters, and they can be in-group or out-group members (Hornsey, 2008). People will perceive others with similarities as insiders or others with distinctions as outsiders (Hogg and Terry, 2000). Similarities affect individuals’ cognitive consistency (Morry, 2005) and subsequent behaviors (Li et al., 2017). Through self-categorizing, individuals’ similarity will stimulate individuals’ self-classification actions and inspire individuals to act as consistently as others in the same groups. Furthermore, self-categorizing will satisfy individuals’ self-enhancement and reduce uncertainty (Hogg and Terry, 2000).

Individuals not only expect to keep consistency with persons who belong to the same categorization but also try to reach consensus on issues (Haslam and Reicher, 2015). Researchers have found that agreeableness was related to cooperation and helping behavior (Graziano et al., 2007). In other words, individuals in the same category will achieve consistency with in-group members, cooperate with and help each other. According to the above theory, we argue that deep-level similarity between subordinates and supervisors may affect subordinate *moqi*.

### Deep-Level Similarity and Subordinate *Moqi*

Similarly, anything that is shared among persons includes surface-level similarity and deep-level similarity (Ndiaye, 2011). Harrison et al. (1998) assert that demographic variables can characterize the surface-level similarity. However, Ndiaye (2011) believes that the deep-level similarity is a common characteristic existing in behaviors or interactions (e.g., attitude, belief, and value similarity). Previous research found that deep-level similarity was a predictor of individuals’ behaviors and work outcomes. When employees are deep-level similarity with their colleagues, they will actively seek feedback (Kammeyer-Mueller et al., 2011) which is related to subordinate *moqi* (Zheng et al., 2019a). We assume that subordinates’ deep-level similarity with supervisors may induce subordinate *moqi*. Deep-level similarity reveals that individuals’ consistencies in belief, value, and attitude significantly affect individuals’ judgment, decision, and action on issues or goals (Ndiaye, 2011). When subordinates have deep-level similarities with their supervisors, they will more tend to maintain consistency in intrinsic ideology and show consistent understanding and action of the goals (Zhang and Zhang, 2011). Therefore, when supervisors convey implicit clues to subordinates, their similarities promote subordinates’ capability to accurately explain these clues, correctly understand the real intentions of supervisors beyond the implicit information, and conduct the same behaviors that satisfy supervisors’ expectations. Additionally, previous research found employees who have deep-level similarity with their colleagues engaged in more proactive feedback-seeking (Kammeyer-Mueller et al., 2011). Existing study found that proactive feedback-seeking is positively related to subordinate *moqi* (Zheng et al., 2019a). According to the above, we infer that the more subordinates are deep-level similarity with supervisors, the more they achieve subordinate *moqi* with supervisors. Thus, we hypothesize:

**Hypothesis 1:** Deep-level similarity is positively related to subordinate *moqi.*

### Deep-Level Similarity and Leader-Member Exchange

Leader-member exchange (LMX) reflects the quality of exchange in relationships between leaders and members (Gomez and Rosen, 2001). Subordinates with low-level LMX relationships generally feel that they are out-group people, while employees with high-level LMX consider themselves as in-group people (Zhao et al., 2014). In-group employees can attain more resources (e.g., information, confidence, and involvement) and achieve good work outcomes (Li et al., 2017), whereas out-group employees have few opportunities (Gomez and Rosen, 2001). In this study, we adopt the self-categorization theory to discuss the relationships between deep-level similarity and LMX.

In this study, deep-level similarity emphasizes the homogeneity in value belief, work type, and work attitude between subordinates and supervisors (Ensher et al., 2002). The self-categorization theory proposes that similarity is a prominent indicator of classification (Hogg and Terry, 2000; Haslam and Reicher, 2015). We assume that deep-level similarity, as an indicator of classification, may stimulate employees with a high level of deep-level similarity with supervisors to classify themselves into the same class as their supervisors, and further increase the interpersonal attractiveness (Zuo and Gao, 2008). As the interpersonal attractiveness increases, employees’ cognitive factors related to the LMX relationship (Dulebohn et al., 2012), such as positive affect, employees’ perceived insider status, and psychological security, will increase (Gonzaga et al., 2007; Li et al., 2017). Moreover, previous studies have provided supportive empirical evidence that actual similarity (e.g., competence, personality, and expectations) was directly associated with LMX (Montoya et al., 2008; Erdogan and Bauer, 2015; Liao and Hui, 2021). According to these clues, we infer that subordinates may build a high-quality LMX relationship when they are deep-level similarity with their supervisors. Thus, we hypothesize:

**Hypothesis 2:** Deep-level similarity is positively related to leader-member exchange.

### Leader-Member Exchange and Subordinate *Moqi*

Both LMX and subordinate *moqi* are relational constructs that reflect the interactive relationships between subordinates and supervisors. However, LMX is mainly based on trust, respect, and obligation (Graen and Uhl-Bien, 1995) and emphasizes an exchange relationship between subordinates and supervisors based on social exchange (Graen and Scandura, 1987). In this relationship, both supervisors and subordinates can be initiators of exchange behaviors, and they can get benefits through LMX. Differently, subordinate *moqi* is mainly based on cognitive consistency (Zhang and Zhang, 2011) and reflects a state of subordinates’ understanding and cooperating with their supervisors when they do not speak with their supervisors (Zheng et al., 2019a). In the subordinate *moqi* context, supervisors are implicit information senders, while subordinates are implicit information capturers and interpreters who can explain implicit information accurately. In a word, subordinate *moqi* emphasizes that subordinates have reached a consistent cognition with their supervisors on information or actions (Zhang and Zhang, 2011).

We believe that LMX may positively help subordinates cultivate a state of *moqi* with their supervisor. The emergence of subordinate *moqi* requires that subordinates are familiar with their supervisors and clearly understand their supervisors. Employees with subordinate *moqi* can correctly understand supervisors’ concealed expectations, intentions, or desires based on implicit information conveyed by supervisors (Zheng et al., 2019a). Subordinates may go through three steps to achieve the state of *moqi* with supervisors. Firstly, they should walk close to supervisors to interact. Then, they try to interact with supervisors to be familiar. Finally, they will be familiar to understand implicit information from supervisors (Zheng et al., 2019a). During this process, interacting with supervisors, as a form of LMX, may determine the success of establishing a state of *moqi* with their supervisor.

When subordinates have high-level LMX, they will acquire a series of positive psychological resources, such as trust, respect, and obligation (Graen and Uhl-Bien, 1995), which will lead subordinates to be more willing to walk close to and support supervisors (Choy et al., 2016; Liao and Hui, 2021). The more subordinates exchange with their supervisors, the more benefits they get, which narrows the psychological distance between subordinates and supervisors, and increases their further interactions (Gomez and Rosen, 2001; Liao and Hui, 2021). As the frequency of interaction increases, subordinates and supervisors become more familiar with each other and “match” with each other. It creates preconditions for subordinates in comprehending supervisors’ ideas unspoken. Furthermore, since employees with a high-quality LMX often have more opportunities to access information related to their jobs (Graen and Scandura, 1987), they may have advantages in forming consistent cognition with supervisors because of the supports of the availability of adequate information for helping them comprehend, judge supervisors’ real intentions, and achieve subordinate *moqi* (Zhang and Zhang, 2011). Finally, subordinates with high-level LMX have stronger self-efficacy and are more likely to explore unknown things (Zhao et al., 2014); therefore, they may be motivated to interpret implicit information conveyed from supervisors and to achieve a state of *moqi* with supervisors (Zheng et al., 2019a). Thus, we hypothesize:

**Hypothesis 3:** Leader-member exchange is positively related to subordinate *moqi*.

### The Mediating Role of Leader-Member Exchange

According to the above discussion, as the degree of subordinates’ deep-level similarity with their supervisor increase, the sense of being insiders of subordinates with supervisors will increase. This sense will enhance the interactions between subordinates and supervisors and the quality of the LMX relationships (Hypothesis 2). The frequent interactions between subordinates and supervisors will make subordinates familiar with their supervisors. As the two parties (i.e., subordinates and supervisors) know each other, subordinates may understand supervisors even if they do not talk to each other. If two parties can keep achieving tacit understanding and agreements in the workplace context, they can achieve, namely *moqi* (Hypothesis 3). Thus, we propose that the influence of deep-level similarity on subordinate *moqi* will be indirectly transmitted via LMX. We hypothesize:

**Hypothesis 4:** Leader-member exchange mediates the positive effect of deep-level similarity on subordinate *moqi*.

## Materials and Methods

### Participants and Procedures

We collected the data from employees of 9 firms in Shanghai, Beijing, and Chongqing municipalities, and Jiangsu, Zhejiang, and Guangdong Provinces. The fields of these firms are electrical appliance sales, information technology, and plastic product production. In each company, we introduced the research content to a manager and invited this manager to assist in distributing the online questionnaire among their colleagues. Every participant is distributed a unique code to match their completed questionnaires at time 1 and at time 2. To control the negative effect of common method variance (Podsakoff et al., 2003), we surveyed two times. In period 1, we invited 523 participants to report their deep-level similarities with their supervisors. One month later, we asked these 523 participants to report LMX and subordinate *moqi* and collected 451 questionnaires. Each participant was rewarded with a red envelope after completing the survey and their responses to the questionnaire were kept confidential. Since some participants did not write correctly their unique code at time 1 and time 2, namely, the code of the second time was not the same as the one of the first time. These questionnaires did not be matched correctly, thus can’t be used. Finally, we got 316 valid data for the study.

Among the final participants, 68% of the participants were males. 35.4% were between 26 and 30 years old, and 46.5% were between 30 and 40 years old. While only 3.8% of respondents were beyond 40, and 14.2% were between 18 and 25 years old. In terms of the time of working with supervisors, 44.6 percent of subordinates worked with their supervisors for 1–3 years. In contrast, 23.1 percent of subordinates worked with their supervisors for less than 1 year, and 32.3 percent of subordinates worked with their supervisors beyond three years.

Additionally, 17.1% respondents have worked less than 1 year, 40.8% for 1–3 years, 12.7% for 3–5 years, 16.1% for 5–8 years and 13.3% for more than 8 years. Finally, 25.9 percent of respondents had Associate’s degrees, 35.1 percent had Bachelor’s degrees, and 34.2 percent of respondents had high school degrees. However, a small group of participants had a Master’s qualification (4.7%).

### Measures

This study used the seven-point Likert scales (from 1 = strongly disagree to 7 = strongly agree) to measure the variables.

#### Subordinate *Moqi*

We assessed subordinate *moqi* using an 8-item scale from Zheng et al. (2019a). One example item was, “without explicit verbal communication or overt cues from my supervisor, I can usually understand his/her any ambiguities and concerns about work.” The Cronbach’s alpha of the scale was 0.93.

#### Deep-Level Similarity

We used an 8-item scale of Ensher et al. (2002) to assess the degree of the deep-level similarity between subordinates and supervisors. One sample of measures was “My mentor and I see things in much the same way.” The Cronbach’s alpha of the scale was 0.93.

#### Leader-Member Exchange

This study adopted a 7-item scale of Scandura and Graen (1984) to measure the LMX. We invited subordinates to independently and objectively report their attitudes and feelings toward leader-member exchange. One example of items was, “Regardless of how much formal authority your immediate supervisor has built into his or her position, what are the chances that he or she would be personally inclined to use power to help you solve problems m your work?” The Cronbach’s alpha of the scale was 0.88.

#### Control Variables

Following previous studies of subordinate *moqi* (Wang et al., 2018; Zheng et al., 2019a), subordinates’ gender, age, education, tenure in the current firm, and time of working with their supervisors are related to fostering *moqi* with their supervisors. Thus, this study controlled these variables.

## Results

### Analytic Strategy

First, we adopted SPSS18 software to conduct descriptive analysis and correlation coefficient analysis among study variables. Then, we used MPLUS7 software to conduct confirmatory factor analyses (CFA) to test the measurements’ discriminant validity. Finally, we applied the Structural Equation Modeling and the bootstrapping approach in MPLUS 7 to test the hypotheses.

### Descriptive Statistics and Correlations

The means, standard deviations and correlations of variables exhibit in Table 1. It shows that subordinate *moqi* is positively correlated with deep-level similarity (*r* = 0.52, *p* < 0.001), and leader-member exchange (*r* = 0.73, *p* < 0.001), respectively. Additionally, Table 1 reveals that the leader-member exchange is positively correlated to deep-level similarity (*r* = 0.54, *p* < 0.001).

**TABLE 1:** Descriptive results and correlations of main variables.

Variables	Mean	*SD*	1	2	3	4	5	6	7
1. Gender	1.32	0.47							
2. Age	3.40	0.79	–0.03						
3. Education	2.10	0.94	–0.06	–0.01					
4. Tenure	2.68	1.30	–0.02	0.42**	0.04				
5. Working time with supervisors	2.33	1.13	–0.08	0.29**	0.05	0.67**			
6. Deep-level similarity	4.97	0.97	−0.18**	0.08	0.07	0.05	0.15**		
7. Leader-member exchange	5.25	0.94	−0.15**	0.08	0.10	0.07	0.11	0.54**	
8. Subordinate *moqi*	5.24	1.04	−0.14**	0.10	0.10	0.07	0.14*	0.53**	0.73**

N = 316. *p < 0.05; **p < 0.01.

### Common Method Deviation Analysis

We adopted two methods to examine the common method bias following the suggestions of Podsakoff et al. (2003). First, this study performed Harman’s single-factor test to diagnose the common method variance. Results showed that three factors were extracted to account for 65.76% of the variance, and the first factor accounted for 47.96% of the variance (less than 50%). Second, we applied the controlling for the effects of an unmeasured latent factor method to ascertain the influence of common method variance. Result showed that the fitness of the model with a latent common methods variance factor (CFI = 0.94; TLI = 0.93; RMSEA = 0.06; SRMR = 0.04) did not obviously outperform the three-factor model without a latent common methods variance factor (CFI = 0.92; TLI = 0.92; RMSEA = 0.07; SRMR = 0.05). Since the change of RMSEA is 0.01, the change of CFI is 0.02, and TLI and SRMR are 0.01, we believe that the common method bias is not serious (Xie and Long, 2008).

### Confirmatory Factor Analysis

We adopted CFA to check the degree of constructs’ distinctiveness. Table 2 shows that three-factor model has a superior fit and good discriminant validity (χ^2^_*df*_ *_=_*_227, N__=__316_ = 534, *p* < 0.001, CFI = 0.92, TLI = 0.92, RMSEA = 0.07, SRMR = 0.05). The three-factor model (M1) better fits the data than alternative models, including two-factor model (M2) that combines leader-member exchange and subordinate *moqi* into one factor (χ^2^_*df*_ *_=_*_229, N__=__316_ = 750, *p* < 0.001, Δχ^2^(2) = 216, *p* < 0.01); two-factor model (M3) that integrates deep-level similarity and LMX into one factor (χ^2^_df_ *_=_*_229, N__=__316_ = 1,085, *p* < 0.001, Δχ^2^(2) = 551, *p* < 0.01); two-factor model (M4) that combines deep-level similarity and subordinate *moqi* into one factor (χ^2^_*df*_ *_=_*_229, N__=__316_ = 1,298, *p* < 0.001, Δχ^2^(2) = 764, *p* < 0.01); and one-factor model (M5) that merges deep-level similarity, leader-member exchange and subordinate *moqi* into one factor (χ^2^_df_ *_=_*_230, N__=__316_ = 1,494, *p* < 0.001, Δχ^2^(3) = 960, *p* < 0.01). Results offer evidence for the good discriminant validity of the variables.

**TABLE 2:** CFA results for the predicting discriminant validity of main variables.

Model	χ ^2^	*d.f.*	Δχ ^2^	RMSEA	CFI	TLI	SRMR
M1: three-factor model	534	227		0.07	0.92	0.92	0.05
M2: two-factor model	750	229	216***	0.09	0.87	0.86	0.06
M3: two-factor model	1,085	229	551***	0.11	0.79	0.77	0.11
M4: two-factor model	1,298	229	764***	0.12	0.74	0.71	0.10
M5: one-factor model	1,494	230	960***	0.13	0.69	0.66	0.11

N = 316. ***p < 0.001. M1 = deep-level similarity, leader-member exchange, subordinate moqi; M2 = deep-level similarity, leader-member exchange + subordinate moqi; M3 = deep-level similarity + leader-member exchange, subordinate moqi; M4 = deep-level similarity + subordinate moqi, leader-member exchange; M5 = deep-level similarity + leader-member exchange + subordinate moqi.

### Hypothesis Testing

**FIGURE 1:**
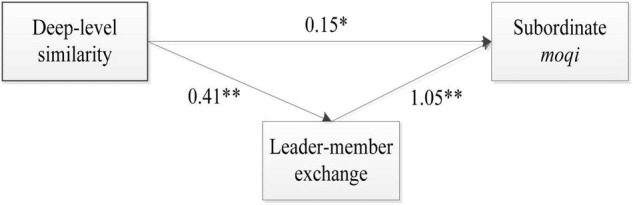
Unstandardized path coefficients from structural equation modeling results. *N* = 316. *p < 0.05; ^**^p < 0.01.

Hypothesis 1 infers that deep-level similarity will significantly predict subordinate *moqi*. Figure 1 shows that the path coefficient between deep-level similarity and subordinate *moqi* is positive and significant (γ = 0.15, *p* = 0.014). Thus, it supports Hypothesis 1.

Hypothesis 2 assumes that deep-level similarity will significantly predict the leader-member exchange. Figure 1 shows that deep-level similarity (γ = 0.41, *p* < 0.001) is significantly correlated to the leader-member exchange. Therefore, it supports Hypothesis 2.

Hypothesis 3 proposes that the LMX is positively associated with subordinate *moqi*. As shown in Figure 1, the path coefficient between the leader-member exchange and subordinate *moqi* is significant (γ = 1.05, *p* < 0.001). Thus, it supports Hypothesis 3.

Hypothesis 4 predicts that LMX mediates the influence of deep-level similarity on subordinate *moqi*. We used a bootstrapping approach to generate the indirect effect coefficient and the confidence intervals of the indirect effects. Table 3 reveals that the indirect influence of deep-level similarity on subordinate *moqi* through the leader-member exchange is significant (γ = 0.43, *p* < 0.001). The 95% confidence interval of the mediating effect is (0.331, 0.567), and correspondingly zero is excluded. Thus, hypothesis 4 is supported.

**TABLE 3:** Mediation effect.

Effect	A	B	Indirect effect	Direct effect	95%CI	
					Lower	Upper
Deep-level similarity - Leader-member exchange - Subordinate *moqi*	0.41**	1.05**	0.43**	0.15*	0.331	0.567

N = 316. *p < 0.05; **p < 0.01.

## Discussion

The present study investigated the relationship between deep-level similarities and subordinate *moqi* from the perspective of self-categorization. Through constructing a mediation model, this study expected to answer whether and how deep-level similarity affects subordinate *moqi*. A two-wave survey was conducted and results showed that subordinates’ deep-level similarities with their supervisors were positively associated with their *moqi* with their supervisors. Moreover, as the degree of subordinates’ deep-level similarities with their supervisors is enhanced, the quality of the leader-member exchange relationship will be higher (Zhang et al., 2013; Liao and Hui, 2021), and in turn, the more subordinates can foster subordinate *moqi*. This study suggested that subordinates’ deep-level similarities with supervisors may enhance their consistent cognition or behavior (Zhang and Zhang, 2011), and then subordinates can exactly guess supervisors’ expectation, intention, and demand unspoken (Zheng et al., 2019a) when they receive clues conveyed through supervisors’ eyes, tone, expressions, and so on. Additionally, our findings suggested that deep-level similarities may inspire subordinates’ self-categorization motivations and regard supervisors, who have deep-level similarities with them, as in-group and then build a high-quality LMX relationship. During the interactive process of LMX, subordinates will have more chances to get information about their supervisors, and then they may improve the level of understanding their supervisors. Subsequently, even without more communication, subordinates also know their supervisors’ real thoughts unspoken; namely, subordinates have built *moqi* with their supervisors. In all, this study shows that deep-level similarity and LMX play important roles in fostering subordinate *moqi* and provides several implications for subordinate *moqi* research.

### Theoretical Implications

First, this study provides researchers and practitioners with a new angle to understand the antecedents of subordinate *moqi*. Previous researchers have discussed the antecedents of subordinate *moqi* from the perspective of subordinates and found that subordinate feedback-seeking (Zheng et al., 2019a) and perceived supervisory status (Wang et al., 2018) could predict subordinate *moqi.* From the perspective of leader, Zhong et al. (2021) examined the effect of leader humility on subordinate *moqi*. However, not enough papers have discussed other antecedents of subordinate *moqi* from the similar traits between subordinates and supervisors’ perspectives. Given that subordinates’ characteristics can influence subordinate *moqi*, similar traits (i.e., deep-level similarity), as a kind of characteristics between subordinates and supervisors, may affect subordinate *moqi*. According to the self-categorization theory, we investigate the influence of deep-level similarity on subordinate *moqi* from the perspective of the connection between subordinates and supervisors. This study provides evidence that the traits linking subordinates and supervisors can affect subordinate *moqi*.

Second, our study provides researchers with a possible path between deep-level similarities on subordinate *moqi*, which serves as a modest spur to induce other researchers to come forward with more contributions. Previous studies have not investigated how the traits linking subordinates and supervisors promote or discourage subordinate *moqi*. From the perspective of self-categorization, when subordinates and supervisors have more deep-level similarities, they may regard themselves as insiders of the same clusters (Haslam and Reicher, 2015). This sense of being in the same groups will enhance the communication between subordinates and supervisors to achieve high-quality LMX relationships (Liao and Hui, 2021), and indirectly promote subordinate *moqi*. Additionally, this study found that deep-level similarities and LMX are positively related to subordinate *moqi*. These findings extend our understanding of subordinate *moqi* from the points of similarities and LMX.

Third, the current research also contributes to followership literature. Previous scholars mainly explore the influencing factors of followers’ behavior from the perspective of a leader or leadership; however, few explore the effect of traits linking subordinates and supervisors on people’s behaviors. This study found that employees’ deep-level similarity with supervisors can induce subordinates’ understandings and cooperation with their supervisors. From the perspective of the traits linking subordinates and supervisors, our findings provide important evidence that subordinates’ similar characteristics (deep-level similarity) with supervisors can play a vital role in influencing subordinates’ behaviors toward their supervisors.

### Managerial Implications

This study proposes several vital implications for organizations. Existing research has provided evidence that subordinate *moqi* can positively influence employees’ task performance and knowledge sharing behavior (Zheng et al., 2019a,b). To adopt the subordinate *moqi* as a tool in management and increase employees’ positive behaviors and performance, organizations may concern the deep-level similarity with supervisors, a predictor of LMX and subordinate *moqi*, and hire employees who have deep-level similarity with supervisors (Kammeyer-Mueller et al., 2011). When HR managers interview candidates, they may evaluate candidates’ values, and working methods through surveys to judge the matching degree of the deep similarity between departments and candidates. Additionally, organizations should cultivate open communication cultures between employees and their supervisors, and provide them with convenient communication channels to help them learn relevant work experience or knowledge from each other. These behaviors can help subordinates and supervisors form deep-level similarities, inspire subordinate *moqi*, and further generate positive outcomes (Zheng et al., 2019a,b; Zhou and Zheng, 2019).

Our study finds that LMX plays an essential role in explaining how employees’ deep-level similarities with their supervisors build subordinate *moqi*. These findings support that if subordinates have high-quality LMX relationships, they will have high-level subordinate *moqi*. Thus, managers should concern more about how to inspire LMX. Firstly, managers should actively seek sufficient resources from organizations for the initiation of LMX. These resources can help supervisors lay the foundation for establishing high-quality LMX (Dulebohn et al., 2012). Secondly, organizations should provide a fair reward system in which employees can receive rewards based on their contribution, which accordingly fosters high-quality LMX relationships (Liao and Hui, 2021). Thirdly, organizations should enhance effective leadership programs training to managers to inspire line managers’ perceptions of LMX and *moqi*.

### Limitations and Future Research

Several limitations in this study should be concerned. First, although we used the time-lag approach to control common method variance rising in the self-reported data in this study, the effect of common method variance cannot be utterly avoided. To better measure the variables and understand the cause-effect of the phenomenon, scholars should collect data from multiple sources and use other research designs (e.g., between-subject design experiment) to investigate the underlying mechanism. Second, although the current study has examined LMX as a mediator in the effects of deep-level similarity on subordinate *moqi*, other variables may also play mediating roles in such relationships. For example, trust-in-supervisor, perceived insider status, and role identity may be possible variables that explain the effect of deep-level similarity on subordinate *moqi*. Thus, future research should test the effects of other mediators to comprehend further how and why deep-level similarity leads to subordinate *moqi*. Third, we collect data only from Chinese firms in this study. Considered high context, face, and high power distance culture are salient in China and are related to fostering *moqi* as well (Zheng et al., 2019a), our findings remain further discussions in generalization in wide cultural contests and industrial contexts.

## Conclusion

Subordinate *moqi* plays a critical role between employees and their supervisors. Though managers recognize the importance of subordinate *moqi*, little is known that how to foster it. Based on the self-categorization theory, this study discusses *whether* and *how* deep-level similarity affects subordinate *moqi*. By analyzing the data from a two-wave survey, we find that deep-level similarity significantly predicts subordinate *moqi* and the leader-member exchange relationships (LMX). Meanwhile, the LMX relationship, enhanced by subordinate *moqi*, further promotes subordinate *moqi*. These findings suggest that deep-level similarity inspires employees’ self-categorization motivations and then builds a high-quality LMX relationship. As subordinates have a high-quality LMX relationship, they have more chances to interact with their supervisor and attain more information about their supervisor and work. Then, employees are more likely to understand their supervisor and build subordinate *moqi*. In other words, this study highlights that deep-level similarity and LMX play imperative roles in fostering subordinate *moqi.* Additionally, this study enriches the literature on the antecedent of subordinate *moqi*, and identifies the influencing mechanism of deep-level similarity in subordinate *moqi*.

## Data Availability Statement

The original contributions presented in the study are included in the article/supplementary material, further inquiries can be directed to the corresponding author/s.

## Ethics Statement

Ethical review and approval was not required for the study on human participants in accordance with the local legislation and institutional requirements. Written informed consent for participation was not required for this study in accordance with the national legislation and the institutional requirements.

## Author Contributions

LL was responsible for the study design, data analysis, drafting, and revising of the manuscript. XZ contributed to the study design, data collection, and offering revising suggestions. SS provided advice and revised the article. All authors approved the submitted version of the article.

## Conflict of Interest

The authors declare that the research was conducted in the absence of any commercial or financial relationships that could be construed as a potential conflict of interest.

## Publisher’s Note

All claims expressed in this article are solely those of the authors and do not necessarily represent those of their affiliated organizations, or those of the publisher, the editors and the reviewers. Any product that may be evaluated in this article, or claim that may be made by its manufacturer, is not guaranteed or endorsed by the publisher.

## Appendix

### Scales and Items

#### Subordinate *Moqi* (Zheng et al., 2019a)

Without explicit verbal communication or overt cues from my supervisor, I can understand his/her task requirements at work.

Without explicit verbal communication or overt cues from my supervisor, I can usually understand any ambiguities and concerns about work for my supervisor.

Without explicit verbal communication or overt cues from my supervisor, I can cooperate with him/her at work.

Without explicit verbal communication or overt cues from my supervisor, I am familiar with my supervisor’s work style.

Without explicit verbal communication or overt cues from my supervisor, I am able to understand his/her train of thought.

Without explicit verbal communication or overt cues from my supervisor, I am clear about my supervisor’s work methods.

Without explicit verbal communication or overt cues from my supervisor, I cooperate well with my supervisor.

Without explicit verbal communication or overt cues from my supervisor, I can cooperate with and act in concert with my supervisor.

## References

[B1] Abu Bakar H. McCann R. M. (2018). Workgroup diversity: surface-level actual similarity and deep-level perceived similarity in leader-member relationship communication. *Corpor. Commun. Int. J.* 23 35–50. 10.1108/CCIJ-03-2017-0012

[B2] Bao Y. Zhou K. Z. Su C. (2003). Face consciousness and risk aversion: do they affect consumer decision-making? *Psychol. Market.* 20 733–755. 10.1002/mar.10094

[B3] Casimir G. Ng Y. N. K. Wang K. Y. Ooi G. (2014). The relationships amongst leader-member exchange, perceived organizational support, affective commitment, and in-role performance: a social-exchange perspective. *Leaders. Organ. Dev. J.* 35 366–385. 10.1108/LODJ-04-2012-0054

[B4] Chen Y. N. Huang X. Tjosvold D. (2008). Similarity in gender and self-esteem for supportive peer relationships: the mediating role of cooperative goals. *J. Appl. Soc. Psychol.* 38 1147–1178. 10.1111/j.1559-1816.2008.00343.x

[B5] Choy J. Mccormack D. Djurkovic N. (2016). Leader-member exchange and job performance: the mediating roles of delegation and participation. *J. Manage. Dev.* 35 104–119. 10.1108/JMD-06-2015-0086

[B6] Deng L. Dai L. Fang X. (2014). The relationship among similarity of couple’s value, communication pattern and marital quality. *Stud. Psychol. Behav.* 12 231–237. 10.1037/0893-3200.20.3.448 16938003

[B7] Dulebohn J. H. Bommer W. H. Liden R. C. Brouer R. L. Ferris G. R. (2012). A meta-analysis of antecedents and consequences of leader-member exchange: integrating the past with an eye toward the future. *J. Manage.* 38 1715–1759. 10.1177/0149206311415280

[B8] Ensher E. A. Grant-Vallone E. J. Marelich W. D. (2002). Effects of perceived attitudinal and demographic similarity on protégés’ support and satisfaction gained from their mentoring relationships. *J. Appl. Soc. Psychol.* 32 1407–1430. 10.1111/j.1559-1816.2002.tb01444.x

[B9] Erdogan B. Bauer T. N. (2015). Leader–member exchange theory. *Int. Encyclo. Soc. Behav. Sci.* 13 641–647. 10.1016/B978-0-08-097086-8.22010-2

[B10] Fisher D. M. Bell S. T. Dierdorff E. C. Belohlav J. A. (2012). Facet personality and surface-level diversity as team mental model antecedents: implications for implicit coordination. *J. Appl. Psychol.* 97 825–841. 10.1037/a0027851 22468847

[B11] Gomez C. Rosen B. (2001). The leader-member exchange as a link between managerial trust and employee empowerment. *Group Organ. Manage.* 26 53–69. 10.1177/1059601101261004

[B12] Gonzaga G. C. Campos B. Bradbury T. (2007). Similarity, convergence, and relationship satisfaction in dating and married couples. *J. Personal. Soc. Psychol.* 93 34–48. 10.1037/0022-3514.93.1.34 17605587

[B13] Graen G. B. Scandura T. A. (1987). Toward a psychology of dyadic organizing. *Res. Organ. Behav.* 9 175–208.

[B14] Graen G. B. Uhl-Bien M. (1995). Relationship-based approach to leadership: development of leader-member exchange (LMX) theory of leadership over 25 years: applying a multi-level multi-domain perspective. *Leaders. Q.* 6 219–247. 10.1016/1048-9843(95)90036-5

[B15] Graziano W. G. Habashi M. M. Sheese B. E. Tobin Renée M. (2007). Agreeableness, empathy, and helping: a person × situation perspective. *J. Personal. Soc. Psychol.* 93 583–599. 10.1037/0022-3514.93.4.583 17892333

[B16] Harrison D. A. Pricem K. H. Bell M. P. (1998). Beyond relational demography: time and the effect of surface-and deep-level diversity on work group cohesion. *Acad. Manage. J.* 41 96–107. 10.2307/256901

[B17] Haslam S. A. Reicher S. D. (2015). Self-categorization theory. *Int. Encyclo. Soc. Behav. Sci.* 21 455–459. 10.1016/B978-0-08-097086-8.24087-7

[B18] Ho Y. F. (1976). On the concept of face. *Am. J. Soc.* 81 867–884. 10.1086/226145

[B19] Hofstede G. Bond M. H. (1984). Hofstede’s culture dimensions: an independent validation using rokeach’s value survey. *J. Cross-Cult. Psychol.* 15 417–433. 10.1177/0022002184015004003

[B20] Hogg M. A. Terry D. J. (2000). Social identity and self-categorization processes in organizational contexts. *Acad. Manage. Rev.* 25 121–140. 10.5465/amr.2000.2791606

[B21] Hornsey M. J. (2008). Social identity theory and self-categorization theory: a historical review. *Soc. Personal. Psychol. Compass* 2 204–222. 10.1111/j.1751-9004.2007.00066.x

[B22] Kammeyer-Mueller J. D. Livingston B. A. Liao H. (2011). Perceived similarity, proactive adjustment, and organizational socialization. *J. Vocational Behav.* 78 225–236. 10.1016/j.jvb.2010.09.012

[B23] Kim J. Y. Sang H. N. (1998). The concept and dynamics of face: implications for organizational behavior in Asia. *Organ. Sci.* 9 522–534. 10.1287/orsc.9.4.522 19642375

[B24] Li L. Zheng X. (2020). The influence of subordinates’ moqi with supervisors on employees’ work engagement: the role of trust-in-supervisor and error aversion culture”. *Hum. Res. Dev. Chin.* 37 57–68. 10.16471/j.cnki.11-2822/c.2020.9.004

[B25] Li L. Zheng X. Sun S. Diaz I. (2020). Does subordinate moqi affect leadership empowerment? *Leaders. Organ. Dev. J.* 41 1015–1034. 10.1108/LODJ-08-2019-0351

[B26] Li X. Liu H. Chen B. (2017). The impacts of deep-level similarity perception with supervisor on the employee’s innovative behavior: a test of two mediating effects. *Sci. Technol. Prog. Policy* 34 146–152.

[B27] Liao E. Y. Hui C. (2021). A resource-based perspective on leader-member exchange: an updated meta-analysis. *Asia Pacif. J. Manage.* 38 317–370. 10.1007/s10490-018-9594-8

[B28] Liao H. Liu D. Loi R. (2010). Looking at both sides of the social exchange coin: a social cognitive perspective on the joint effects of relationship quality and differentiation on creativity. *Acad. Manage. J.* 53 1090–1109. 10.5465/amj.2010.54533207

[B29] Liao J. Q. Zhao J. Zhang Y. J. (2010). The influence of power distance on leadership behavior in China. *Chin. J. Manage.* 7 988–992.

[B30] Montoya R. M. Horton R. S. Kirchner J. (2008). Is actual similarity necessary for attraction? A meta-analysis of actual and perceived similarity. *J. Soc. Pers. Relat.* 25 889–922. 10.1177/0265407508096700

[B31] Morry M. M. (2005). Relationship satisfaction as a predictor of similarity ratings: a test of the attraction-similarity hypothesis. *J. Soc. Personal. Relat.* 22 561–584. 10.1177/0265407505054524

[B32] Ndiaye M. (2011). *The Impact of Deep-Level Similarity on Career Advancement Intentions Among High Level executives in Athletics.* Storrs: University of Connecticut, USA. [Ph.D.Thesis].

[B33] Oldmeadow J. Platow M. Foddy M. Anderson D. (2003). Self-categorization, status, and social influence. *Soc. Psychol. Q.* 66 138–152. 10.2307/1519844

[B34] Podsakoff P. M. Mackenzie S. B. Lee J. L. Podsakoff N. P. (2003). Common method biases in behavioural research: a critical review of the literature and recommended remedies. *J. Appl. Psychol.* 88 879–903. 10.1037/0021-9010.88.5.879 14516251

[B35] Richardson R. M. Smith S. W. (2007). The influence of high/low-context culture and power distance on choice of communication media: students’ media choice to communicate with Professors in Japan and America. *Int. J. Intercult. Relat.* 31 479–501. 10.1016/j.ijintrel.2007.01.002

[B36] Scandura T. A. Graen G. B. (1984). Moderating effects of initial leader-member exchange status on the effects of a leadership intervention. *J. Appl. Psychol.* 69 428–436. 10.1037/0021-9010.69.3.428

[B37] Sherony K. M. Green S. G. (2002). Coworker exchange: relationships between coworkers, leader-member exchange, and work attitudes. *J. Appl. Psychol.* 87 542–548. 10.1037/0021-9010.87.3.542 12090611

[B38] Srivastava A. P. Dhar R. L. (2016). Impact of leader member exchange, human resource management practices and psychological empowerment on extra role performances: the mediating role of organisational commitment. *Int. J. Productiv. Performan. Manage.* 65 351–377. 10.1108/IJPPM-01-2014-0009

[B39] Valenzuela M. A. Jian G. Jolly P. M. (2020). When more is better: the relationships between perceived deep-level similarity, perceived workplace ethnic diversity, and immigrants’ quality of coworker relationships. *Employ. Relat.* 42 507–524. 10.1108/ER-05-2019-0202

[B40] Wang L. Ye M. Chen Y. Wang Z. (2018). The effect of perceived supervisory status on subordinates’ moqi-the roles of feedback seeking behavior and perspective taking. *J. Psychol. Sci.* 41 1200–1206. 10.16719/j.cnki.1671-6981.20180526

[B41] Xie B. L. Long L. R. (2008). The effects of career plateau on job satisfaction, organizational commitment and turnover intentions. *Acta. Psychol. Sin.* 40 927–938. 10.1002/nop2.872 33826252 PMC8363357

[B42] Yao J. S. Chen G. (2017). Creating a green context: a study of media strategies in the cultivating moqi of environmental governance. *J. Capit. Normal Univers. Soc. Sci. Ed.* 237 143–148.

[B43] Zhang K. Z. Zhang Q. Y. (2011). Discuss the consensus and moqi in joint action. *Tianjing Soc. Sci.* 5 58–67. 10.16240/j.cnki.1002-3976.2011.05.015

[B44] Zhang X. A. Cao Q. Grigoriou N. (2011). Consciousness of social face: the development and validation of a scale measuring desire to gain face versus fear of losing face. *J. Soc. Psychol.* 151 129–149. 10.1080/00224540903366669 21476458

[B45] Zhang Z. Wang M. Shi J. (2013). Leader-follower congruence in proactive personality and work outcomes: the mediating role of leader-member exchange. *Acad. Manage. J.* 55 111–130. 10.5465/amj.2009.0865

[B46] Zhao H. Kessel M. Kratzer J. (2014). Supervisor-subordinate relationship, differentiation, and employee creativity: a self-categorization perspective. *J. Creativ. Behav.* 48 165–184. 10.1002/jocb.46

[B47] Zheng X. Li N. Harris T. B. Liao H. (2019a). Unspoken yet understood: an introduction and initial framework of subordinates’ moqi with supervisors. *J. Manage.* 45 955–983. 10.1177/0149206316687642

[B48] Zheng X. Li L. Zhang F. Zhu M. (2019b). The roles of power distance orientation and perceived insider status in the subordinates’ moqi with supervisors and sustainable knowledge-sharing. *Sustainability* 11:1421. 10.3390/su11051421

[B49] Zhong J. Zhang L. Xiao H. Wen Q. (2021). Antecedents and consequences of follower moqi: leader humility, follower humility, and knowledge hiding. *Curr. Psychol.* 2021 1–12. 10.1007/s12144-021-02001-1

[B50] Zhou X. Zheng X. (2019). The relationship between subordinate’ moqi and supervisor’s Overall Management Evaluation: the Mediating Effect of Voice and the Moderating Effect of Personal Power Distance Orientation. *Shanghai Manage. Sci.* 41 52–56.

[B51] Zuo B. Gao Q. (2008). The effects of familiarity and similarity on the interpersonal attraction. *Chin. J. Clin. Psychol.* 19 633–636. 10.16128/j.cnki.1005-3611.2008.06.027

